# The IL6-174G/C Polymorphism Associated with High Levels of IL-6 Contributes to HCV Infection, but Is Not Related to HBV Infection, in the Amazon Region of Brazil

**DOI:** 10.3390/v14030507

**Published:** 2022-02-28

**Authors:** Maria Alice Freitas Queiroz, Angélica Menezes Santiago, Tuane Carolina Ferreira Moura, Ednelza da Silva Graça Amoras, Simone Regina Souza da Silva Conde, Izaura Maria Vieira Cayres-Vallinoto, Ricardo Ishak, Antonio Carlos Rosário Vallinoto

**Affiliations:** 1 Laboratory of Virology, Institute of Biological Sciences, Federal University of Pará (UFPA), Belém 66075-110, Brazil; angel_ams3@hotmail.com (A.M.S.); tuanecfmoura@gmail.com (T.C.F.M.); ednelza@hotmail.com (E.d.S.G.A.); ivallinoto@ufpa.br (I.M.V.C.-V.); rishak@ufpa.br (R.I.); vallinoto@ufpa.br (A.C.R.V.); 2 Graduate Program in Virology, Evandro Chagas Institute/SVS/MS, Rodovia BR-316 km 7 s/n, Ananindeua 67030-000, Brazil; 3 Graduate Program in Biology of Infectious and Parasitic Agents, Institute of Biological Sciences, Federal University of Pará (UFPA), Belém 66075-110, Brazil; 4 Pathology Service, João de Barros Barreto University Hospital, Federal University of Pará (UFPA), Belém 66073-000, Brazil; sconde@ufpa.br; 5 Institute of Health Sciences, School of Medicine, Federal University of Pará (UFPA), Umarizal, Belém 66075-110, Brazil

**Keywords:** HBV, HCV, chronic hepatitis, polymorphism, IL-6, plasma dosage

## Abstract

The dysregulation of cytokine production can lead to an inefficient immune response, promoting viral persistence that induces the progression of chronic viral hepatitis. The study investigated the association of the IL6-174G/C polymorphism with changes in cytokine levels and its influence on the persistence and progression of chronic hepatitis caused by HBV and HCV in 72 patients with chronic hepatitis B (HBV), 100 patients with hepatitis C (HCV), and a control group of 300 individuals. The genotyping of the IL6-174G/C polymorphism was performed by real-time PCR, and cytokine levels were measured by enzyme-linked immunosorbent assay (ELISA). HCV patients with the wild-type genotype (GG) had a higher viral load (*p* = 0.0230). The plasma levels of IL-6 were higher among patients infected with HBV and HCV than among the control group (*p* < 0.0001). Patients with HCV were associated with increased inflammatory activity (A2–A3; *p* < 0.0001). In hepatitis C, carriers of the GG genotype had higher levels of IL-6 (*p* = 0.0286), which were associated with A2–A3 inflammatory activity (*p* = 0.0097). Patients with A2–A3 inflammatory activity and GG genotype had higher levels of IL-6 than those with the GC/CC genotype (*p* = 0.0127). In conclusion, the wild-type genotype for the IL6-174G/C polymorphism was associated with high levels of IL-6 and HCV viral load and inflammatory activity, suggesting that this genotype may be a contributing factor to virus-induced chronic infection.

## 1. Introduction

Hepatitis C virus (HCV) and hepatitis B virus (HBV) are the two main infectious agents that cause hepatitis worldwide. It is estimated that 296 million people are chronically infected with HBV worldwide, while 71 million are chronic HCV carriers [1]. These viruses cause liver inflammation, which can be persistent. Chronic HBV and HCV infections represent a major health problem because they can progress to liver failure, liver cirrhosis, and hepatocellular carcinoma [2,3].

In chronic infection, the dysregulation of host immunological factors leads to an inefficient immune response, promoting viral persistence and influencing the immunopathogenesis of HBV- and HCV-induced hepatitis [4,5]. The altered production of cytokines is an important factor that can contribute to the outcome of hepatitis B and hepatitis C [6,7].

IL-6 promotes the activation of hepatocytes and immunocompetent and hematological cells, which induce immunological and hematological responses. These responses are essential to fight and eliminate infection; therefore, IL-6 is essential for host defense [8]. 

Changes in IL-6 transcription levels have been related to the presence of several polymorphisms in the promoter region of the gene [9]. The IL6-174G/C polymorphism was shown to be associated with variations levels of circulating IL-6; the CC genotype was related to low levels of the cytokine, while the GG genotype showed higher levels of IL-6 [10].

Elevated IL-6 levels during the period of infection or tissue damage can lead to the development of serious complications, while persistent IL-6 expression can induce the onset of chronic inflammatory diseases [8]. The development of severe inflammatory manifestations caused by viral infections has been shown to be associated with high levels of IL-6 [11,12,13]. It is possible that the damage caused to the liver by the virus induces an increase in IL-6 levels, in an attempt to regenerate liver cells, which characterizes the cytokine as hepatoprotective [14]. However, high levels of IL-6 in the presence of HBV and HCV can contribute to the inflammatory process and the development of fibrosis, characterized by extracellular matrix deposition, parenchymal cell death, angiogenesis, and tissue remodeling [15,16,17,18].

In hepatitis B, the influence of polymorphisms in the progression of infection is not yet well defined [19,20], whereas in hepatitis C, studies show a relationship between the wild-type allele and disease progression [21,22]. In this context, the objective of this study was to investigate the association of the IL6-174G/C polymorphism with changes in cytokine levels and its influence on the levels of inflammation, fibrosis, and the viral load of HBV and HCV.

## 2. Materials and Methods

### 2.1. Study Design and Population

This cross-sectional study was conducted in the liver disease outpatient clinic of João de Barros Barreto University Hospital and Santa Casa de Misericórdia do Pará Foundation Hospital, selecting consecutive cases of patients with chronic HBV and HCV.

All selected patients were clinically evaluated and subjected to further examination and were subsequently divided into two groups. The first group consisted of 72 patients with chronic hepatitis B and the second group consisted of 100 patients with chronic hepatitis C. All patients are residents of the northeast region of the Amazon and have a tri-hybrid ethnic composition, with genetic contributions from Europeans, Africans, and indigenous peoples [23].

The inclusion criteria adopted for the individuals were as follows: age 18 or older, and HBsAg positivity and/or HBeAg positivity with clinical and liver test abnormalities (chronic hepatitis B group) or positivity for anti-HCV for more than 6 months and positivity for HCV RNA, as criteria for chronic HCV infection [24] with clinical and liver test abnormalities (chronic hepatitis C group) and without antiviral therapy. Individuals coinfected with hepatitis B virus (HBV), hepatitis delta virus, or human immunodeficiency virus (HIV), and patients who used or were using specific antiviral therapy against HCV were excluded from the study.

A control group composed of 300 volunteer blood donors from HEMOPA (Foundation Center for Hemotherapy and Hematology of Pará), seronegative for HBV, HCV, HIV, HTLV, Chagas disease, and syphilis, was used to compare the frequencies of the polymorphism.

### 2.2. Genotyping of IL6-174G/C (rs1800795)

DNA was extracted from peripheral blood leukocytes using a Puregene kit (Gentra Systems, Minneapolis, MI, USA) according to the manufacturer’s protocol, which included cell lysis, protein precipitation, DNA precipitation, and DNA hydration.

Genotyping of the IL6-174G/C polymorphism was performed using real-time polymerase chain reaction (qPCR) in a StepOne PLUS Sequence Detector (Applied Biosystems, Foster City, CA, USA). The predesigned assay (C__1839697_20) (Thermo Fisher, Carlsbad, CA, USA) used for the polymorphism contained a pair of primers and a pair of probes using VIC and FAM labeling for each of the polymorphism alleles. For each reaction, TaqMan^®^ Universal PCR Master Mix (2X), TaqMan^®^ Assay (20X), and 20 µL of DNA were used in a final reaction volume of 10 mL. The following temperature cycles were used in each amplification reaction: 60 °C for 30 s, 95 °C for 10 min, and 50 cycles of 92 °C for 30 s and 60 °C for 1 min and 30 s.

### 2.3. Laboratory Data

Information on the serology of viral liver diseases, liver enzyme levels, histopathology, and plasma viral loads were obtained from updated clinical records. These data were organized in a restricted-access spreadsheet, used only to obtain information related to the objectives of the study.

The histopathological diagnosis followed the French METAVIR classification [25], and the activity of the portal and periportal inflammatory infiltrate was classified from 0 to 3 (A0–A3), with “A0–A1” indicating absent to mild inflammation and “A2–A3” indicating moderate to severe inflammation. The structural changes in the liver parenchyma (degree of fibrosis) were classified from 0 to 4 (F0–F4), with “F0–F1” indicating absent to mild liver fibrosis, “F2” indicating moderate liver fibrosis, and “F3–F4” indicating advanced liver fibrosis or cirrhosis. All data regarding the histopathological profile were obtained from the medical records of the patients.

### 2.4. Quantification of Plasma IL-6 Levels

The plasma IL-6 concentration was measured using an ELISA immunoassay (IL-6 Human ELISA Kit, High Sensitivity, ThermoFisher, Camarillo, CA, USA). This method uses specific monoclonal antibodies to detect cytokines and was performed according to the manufacturer’s instructions.

### 2.5. Statistical Analysis

The information obtained was entered into a database in the Microsoft Office Excel 2013 software. The calculation of the Hardy–Weinberg balance was performed to assess the distribution of genotypic frequencies. The determination of the allelic and genotypic frequencies of the polymorphisms was made by direct counting and the differences between the groups were evaluated using the chi-squared test and the G test. The distribution of HCV viral load levels and biochemical markers between the genotypes were performed using the Shapiro–Wilk test. From the results of the normality test, non-parametric tests were used (Mann–Whitney test and Kruskal–Wallis test). Statistical analyses were performed with BioEstat software version 5.3, adopting a significance level of *p* < 0.05.

## 3. Results

Most patients in the HBV group were male (*n* = 44; 61.1%). The median levels of the liver enzymes ALT, AST, and GGT were 29, 29, and 33.5 IU/L, respectively, and the viral load (in log10) was 2.9. In the HCV group, most patients were also male (*n* = 52; 52%), with median ALT, AST, and GGT enzyme levels of 60, 64, and 71 IU/L, respectively, and a viral load (in log10) of 5.6 (Table 1).

According to the METAVIR classification (Table 1), most patients with HBV were classified as having absent to moderate fibrosis, F0–F2 (*n* = 60; 83.3%), and absent or mild inflammatory activity, A0–A1 (*n* = 57; 86.4%). In the HCV group, the highest frequency of patients was classified as having absent to moderate fibrosis scores, F0–F2 (*n* = 66; 66%), and absent or mild inflammatory activity, A0–A1 (*n* = 57; 86.4%). Inflammatory activity was evaluated only in 64 HBV and 89 HCV patients, as the other patients were diagnosed with liver cirrhosis through imaging tests and therefore did not meet the criteria for biopsy.

The analysis of Hardy–Weinberg equilibrium showed that the distributions of the genotype frequencies of IL6-174G/C were in equilibrium in the studied groups. The evaluation of the IL6-174G/C polymorphism showed that there were no statistically significant differences between the genotype and allele frequencies in the investigated groups (Table 2).

The analyses of inflammatory activity and fibrosis score related to the IL6-174G/C polymorphism showed that in the HBV and HCV groups, there were no significant differences between the genotype and allele frequencies (Table 3).

Due to the small number of individuals with a homozygous polymorphic genotype for IL6-174G/C and the fact that IL-6 levels are influenced by the presence of at least one polymorphic allele, we evaluated the plasma viral load and plasma IL-6 levels among individuals with the wild-type genotype (GG) and compared them with those among individuals with polymorphic genotypes (GC and CC).

**Figure 1:**
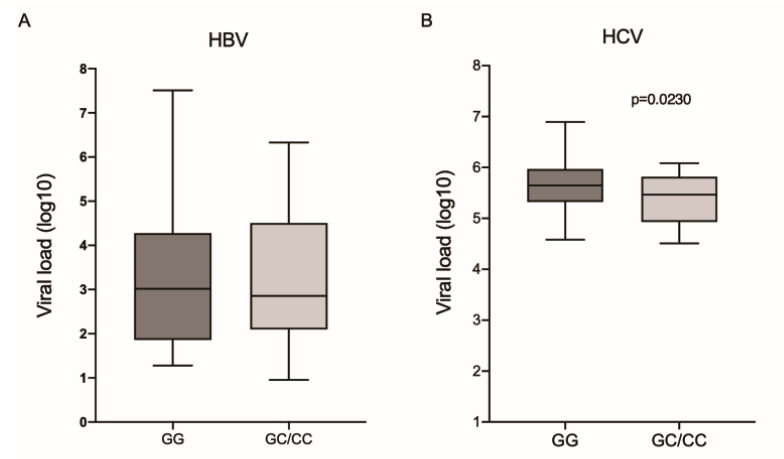
Viral load among the different genotypes for the IL6-174G/C polymorphism in patients with (**A**) HBV and (**B**) HCV. Mann–Whitney test.

The comparison of viral loads showed no difference between the different IL6-174G/C genotypes in the HBV group (Figure 1A). However, HCV patients with the wild-type genotype had a higher viral load (*p* = 0.0230; Figure 1B).

**Figure 2:**
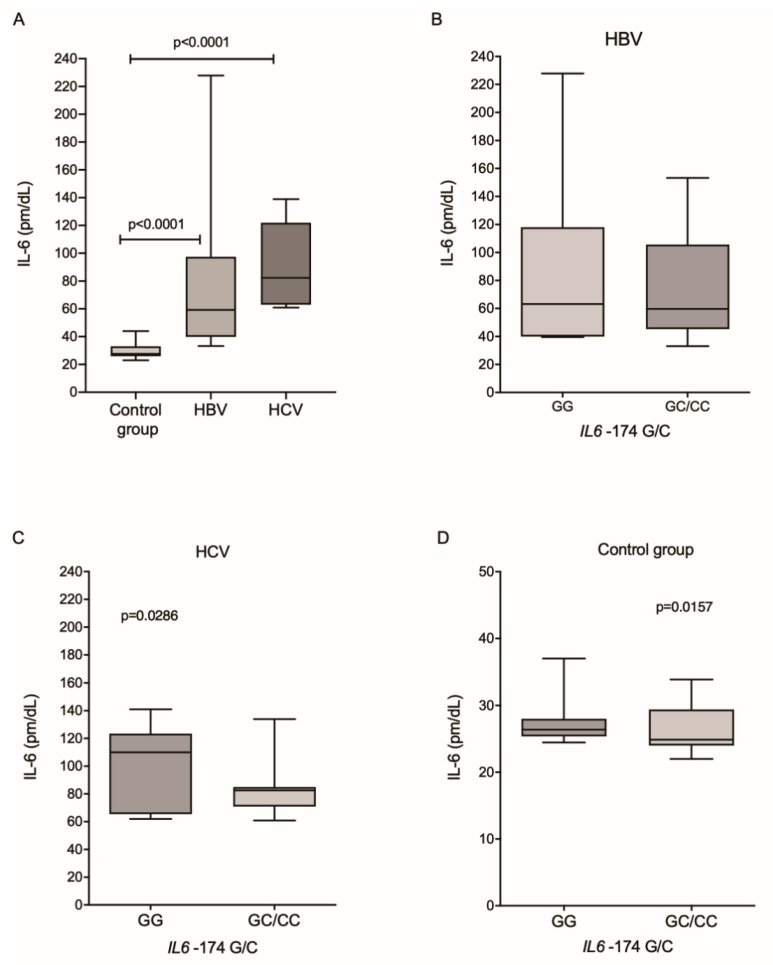
Plasma levels of IL-6 among (**A**) the investigated groups and between the different genotypes for the IL6-174G/C polymorphism in patients with (**B**) HBV and (**C**) HCV and (**D**) in the control group. Kruskal–Wallis test and Mann–Whitney test.

The plasma IL-6 levels were higher among patients infected with HBV and HCV than in the control group (*p* < 0.0001 and *p* < 0.0001, respectively; Figure 2A). In the HBV group, there was no difference in IL-6 levels between IL6-174G/C genotypes (Figure 2B). In contrast, patients from the HCV group (Figure 2C) and control individuals (Figure 2D), who had the wild-type genotype, had higher levels of cytokines (*p* = 0.0286 and *p* = 0.0157, respectively).

**Figure 3:**
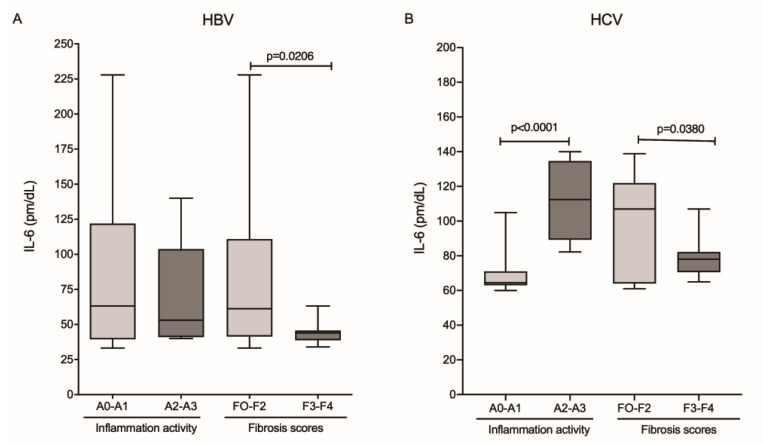
Plasma levels of IL-6 and the degree of inflammatory activity and fibrosis score in the (**A**) HBV and (**B**) HCV groups. Mann–Whitney test.

The analysis of IL-6 levels relative to histopathological markers of fibrosis and inflammatory activity showed that patients in the HBV group with absent-to-mild inflammation (A0–A1) and absent-to-moderate fibrosis scores (F0–F2) had higher IL-6 levels, but those results were significant only for the fibrosis score (*p* = 0.0206; Figure 3A). In the HCV group, cytokine levels were significantly higher in patients with higher inflammatory activity (A2–A3; *p* ≤ 0.0001) and lower in patients with higher fibrosis scores (F3–F4; *p* = 0.0380; Figure 3B).

**Figure 4:**
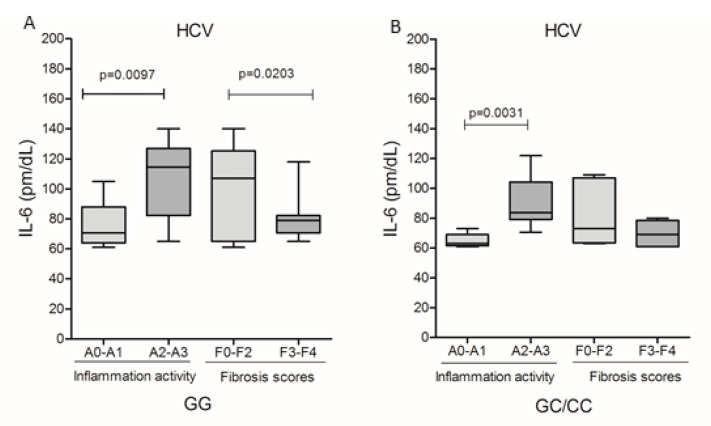
Plasma levels of IL-6 and the degree of inflammatory activity and fibrosis score among carriers of genotypes (**A**) GG and (**B**) GC/CC in HBV infection and patients with genotype (**A**) GG and (**B**) GC/CC infected by HCV. Mann–Whitney test.

Due to the reduced number of patients with HBV who presented histopathological results of inflammatory activity and fibrosis scores, it was not possible to evaluate these markers in relation to the IL6-174G/C polymorphism genotypes. In HCV infection, it was observed that IL-6 levels were higher in patients with A2–A3 inflammatory activity, both in GG (*p* = 0.0097; Figure 4A) and GC/CC (*p* = 0.0031; Figure 4B) genotypes; however, patients with the GG genotype had significantly higher levels of IL-6 compared to patients with the GC/CC genotype (medians 114.5 and 83.63, respectively; *p* = 0.0127). Cytokine levels were higher in patients without fibrosis or who were in early stages of fibrotization (F0–F2) in carriers of the GG genotype (*p* = 0.0203, Figure 4A).

**Figure 5:**
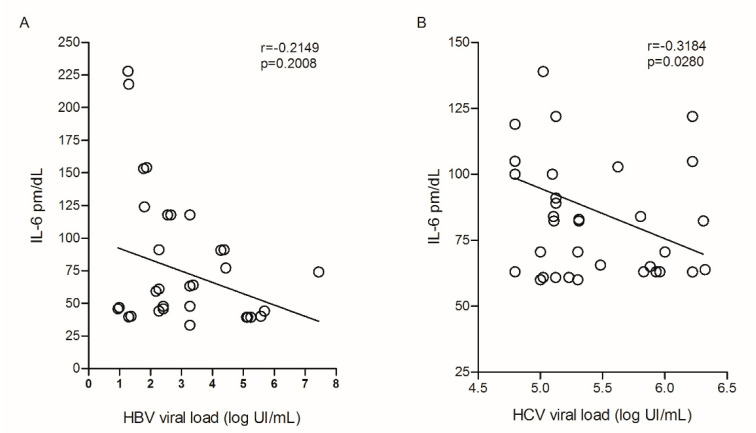
Correlation between plasma IL-6 levels and (**A**) HBV and (**B**) HCV viral load. Spearman test.

The assessment of plasma IL-6 levels with HBV viral load did not show a significant correlation (Figure 5A); however, cytokine levels in relation to HCV viral load showed a negative correlation (r = −0.3184; *p* = 0.0280; Figure 5B).

**Figure 6:**
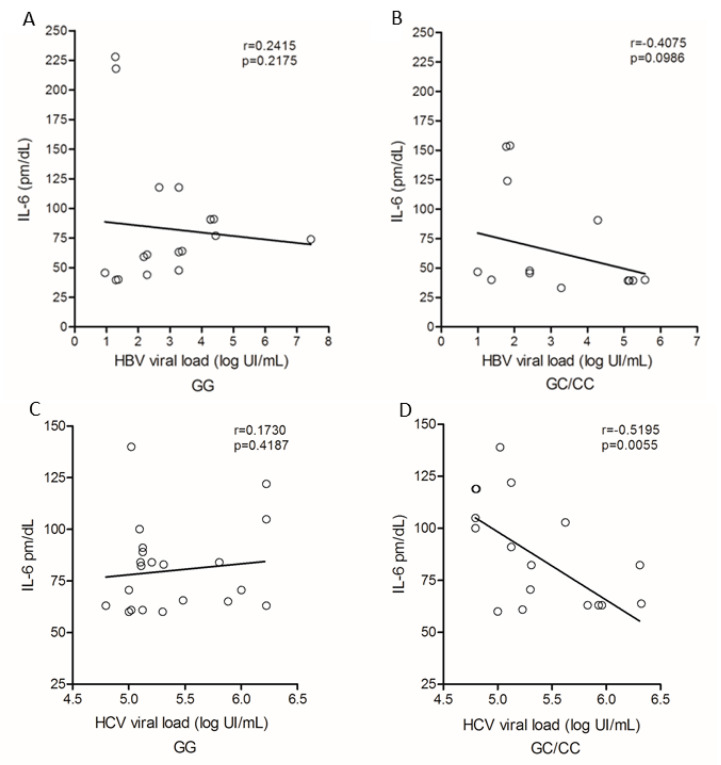
Correlation of plasma IL-6 levels and viral load among carriers of genotypes (**A**) GG and (**B**) GC/CC in HBV infection and patients with genotype (**C**) GG and (**D**) GC/CC infected by HCV. Spearman test.

IL-6 levels and HBV viral load showed no correlation with regard to genotypes for the IL6-174G/C polymorphism (Figure 6A,B). Among patients with chronic hepatitis C, no correlation was observed between cytokine levels and HCV viral load in carriers of the GG genotype (Figure 6C); in contrast, patients with GC/CC genotypes showed a negative correlation (r = −0.5196; *p* = 0.0055; Figure 6D).

## 4. Discussion

Chronic viral infections are induced by active replication of the virus, such as infections caused by HBV and HCV. These types of infections affect the immune response because they induce and maintain altered levels of proinflammatory or immunomodulatory cytokines, which promote the differentiation of the lymphocyte population and induce an increase in specific immune responses [26].

IL-6 is a pleiotropic cytokine involved in several processes of innate immunity and contributes to the differentiation of lymphocytes in cell-mediated immunity [8]. Therefore, altered levels of cytokines may contribute to the dysregulation of the inflammatory immune balance, favoring the development of chronic HBV and HCV infections [27].

The IL6-174G/C polymorphism has been shown to be associated with changes in IL-6 levels under different pathological conditions [10,15,28,29]. In the present study, no association was identified between the frequency of the polymorphism and chronic HBV and HCV infection. The association of the IL6-174G/C polymorphism with chronic HBV infection is not well defined because although several studies have found no association [19,30], some studies suggest that the wild-type (G) genotype is associated with susceptibility to chronic HBV infection [20,31]. In HCV infection, the progression of infection has been shown to be associated with the presence of the G allele [21], and both the GG genotype and the G allele have been shown to be associated with chronic hepatitis C-induced hepatocarcinoma [22]. The differences in the results between these studies may be related to the genetic contribution of the populations studied because these studies were conducted in different ethnic groups. Thus, it is suggested that the frequency of the IL6-174G/C polymorphism may not be directly related to the development of hepatitis B and C in the population of this study, which has a tri-hybrid composition (with genetic contributions from white, black, and indigenous peoples) [23].

Although no association was observed between viral load levels and the IL6-174G/C polymorphism in chronic HBV infection, patients with chronic hepatitis C carrying the GG genotypes had significantly higher viral load levels. No published data on the relationship between IL6-174G/C polymorphism and viral loads in HCV infection were observed. Thus, these results seem to suggest that the wild-type GG allele for the IL6-174G/C polymorphism may favor HCV infection. Other studies observed an association of the GG genotype with the risk for chronic hepatitis C [22,32,33].

IL-6 mediates cellular and humoral immune responses; therefore, adequate cytokine levels are crucial for determining the outcome of viral infection [34]. HBV induces high IL-6 expression, contributing to severe liver injury [35]. High levels of IL-6 contribute to the maintenance of infection because they possibly inhibit apoptosis of infected hepatocytes and control of the infection [36,37]. In the present study, it has been shown that IL-6 levels can be altered by HBV infection, but it was not possible to identify an association between the IL6-174G/C polymorphism and cytokine levels, as observed for viral load. In addition, higher levels of cytokines were observed in patients with milder degrees of infection, as determined by the METAVIR scale. These data suggest that the establishment of chronic hepatitis B may be favored by elevated levels of IL-6; however, no association was observed with the IL6-174G/C polymorphism in the population evaluated. Viral factors seem to favor the establishment of the disease and possibly contribute to the progression of the infection [35].

Although the frequency of the IL6-174G/C polymorphism was not identified as associated with HBV infection, or with variations in IL-6 levels and viral load in the present study, the homozygous polymorphic (CC) genotype was associated with reduced risk for the development of HBV infection, while the GG genotype was associated with increased risk among Iraqi patients [38]. The meta-analysis study performed by Wang et al. (2019) showed that the GC genotype represented a risk for hepatitis C. In contrast, in hepatitis B, both the GG genotype and the association of the GG + GC genotype were identified with the risk for the development of the disease [39]. The association of genotypes with risk for hepatitis B and C seems to depend on the population evaluated. On the other hand, in the present study, the GG genotype was associated with increased levels of IL-6 and viral load in HCV infection, contributing to chronic infection. These results suggest that the impact of the IL6-174G/C polymorphism on viral hepatitis may be different depending on the ethnic contribution of the population.

In HCV infection, the interaction of viral proteins with inefficient T cells results in a rapid increase in viral load and promotes conditions for the exhaustion of the adaptive response and an insufficient innate immune response, contributing to viral persistence in chronically infected patients [40,41]. High levels of IL-6 are associated with HCV infection and, especially, with the most severe forms of hepatitis C [17,42,43]. In the present study, wherein patients with chronic HCV had higher plasma IL-6 levels than did the controls, it was observed that the wild-type genotype for the IL6-174G/C polymorphism may contribute to the increase in cytokine levels. The increase in IL-6 levels is probably one of the immunological mechanisms activated to control HCV infection, as individuals with higher levels of the cytokine have lower levels of viral load, but this does not seem to be enough to prevent the progression of the disease, since IL-6 does not trigger the best response against HC [44]. These results suggest that IL-6 may favor HCV infection and contribute to the development of more severe forms of chronic hepatitis C, as cytokine levels were higher in patients with more pronounced necroinflammatory activity on the METAVIR scale. 

In the evaluation of histopathological markers in relation to HCV infection, an association of the GG genotype with inflammation was identified. IL-6 levels were higher in patients with A2–A3 inflammatory activity, both in GG and GC/CC genotypes; however, patients with the GG genotype had 27% higher cytokine levels than those with the GC/CC genotypes. Furthermore, IL-6 levels were higher in patients without fibrosis or who were in the initial stages of fibrotization (F0–F2) in carriers of the GG genotype, suggesting that this genotype may contribute to accentuating inflammation in the early stages of the disease.

It is important that the disease is diagnosed in the early stages, because once cirrhosis is established, it is more difficult to reverse this process and its complications [45], even in cases where viral clearance occurs. For example, the use of newer treatments against HCV, consisting of direct-acting antivirals (DDAs), promote a sustained virological response, leading to resolution of the HCV infection. However, although viremia eradication improves liver function, this seems not to be sufficient to completely reverse the cellular profile and production of cytokines, including IL-6, in patients with chronic disease, probably as a result of the change in the structure of the liver parenchyma that makes it impossible to recover homeostasis [46,47]. 

The wild-type genotype of the IL6-174G/C polymorphism seems to be a factor that contributes to the infection of HCV, because in addition to being associated with increased IL-6 levels, it was also related to increased viral load. IL-6 promotes the differentiation of CD4+ T cells into Th17 cells and inhibiting Treg differentiation [48,49]. However, despite IL-6 acting as a potent pro-inflammatory cytokine on T cells by promoting Th17 differentiation, these subpopulations of T cells do not represent the most effective type of response in combating HCV and may favor the persistence of infection. Thus, the GG genotype may be considered an important factor that contributes to intensify the inflammatory process of chronic hepatitis C.

Despite the importance of the results found in the present study, this one has a limitation due to the sample size evaluated, which may have interfered in some associations, mainly related to chronic HBV infection.

## 5. Conclusions

Elevated IL-6 levels have been associated with chronic HBV infection, but the IL6-174G/C polymorphism does not appear to influence the disease. In contrast, the wild-type genotype for the IL6-174G/C polymorphism was linked to high IL-6 levels and HCV viral load, suggesting that this genotype may be a contributing factor to the development of chronic hepatitis C in patients from the Amazon region.

## Figures and Tables

**Table 1:** Clinical, laboratory, and histopathological characterization of the group evaluated with chronic hepatitis B and hepatitis C.

Variables	HBV (*n* = 72)	HCV (*n* = 100)
Sex, F/M (%)	28 (38.9)/44 (61.1)	48 (48)/52 (52)
ALT (UI/L), median/IQR	29/35.5	60/62.5
AST (UI/L), median/IQR	29/29.5	64/55.5
GGT (UI/L), median/IQR	33.5/34.3	71/91
Viral load (log 10), median/IQR	2.9/2.4	5.6/0.6
Fibrosis score		
0 a 2	60 (83.3%)	66 (66%)
3 a 4	12 (16.7%)	34 (34%)
Inflammatory activity	*	**
0 a 1	55 (86.4%)	53 (60%)
2 a 3	9 (13.6%)	36 (40%)

ALT: alanine aminotransferase (reference value: 16–40 IU/L); AST: aspartate aminotransferase (reference value: 08-54 IU/L); GGT: gamma-glutamyl transferase (reference value: 08–63 IU/L). Fibrosis score METAVIR: 0: absence of septa; 1: portal fibrosis without septa; 2: portal fibrosis with rare septa; 3: numerous septa but without cirrhosis; 4: cirrhosis. Inflammatory activity: 0: absence of activity; 1: minimum activity; 2: moderate activity; 3: intense activity. IQR: interquartile range. * *n* = 64; ** *n* = 89.

**Table 2:** Allele and genotype frequencies of IL6-174G/C polymorphisms in patients with chronic hepatitis B and chronic hepatitis C and in the control group.

Genotypic and Allelic Profile	HBV * n (%)	HCV ** n (%)	Control *n* (%)	p1	p2
GG	51 (70.8%)	72 (72%)	207 (69%)	0.9488 ^g^	0.5519 ^g^
GC	19 (26.4%)	27 (27%)	85 (28.3%)		
CC	2 (2.8%)	1 (1%)	8 (2.7%)		
G	0.84	0.86	0.83	0.9760 ^c^	0.8232 ^c^
C	0.16	0.14	0.17		

*n*: number of individuals; g: G test; c: chi-squared test. Patients without genotyping: * *n* = 2 and ** *n* = 1.

**Table 3:** Evaluation of the association of the IL6-174G/C polymorphism with inflammatory activity and fibrosis score in chronic HBV and HCV carriers.

Genetic Profile	Inflammatory Activity			Fibrosis Score		
	0 to 1	2 to 3	*p*	0 to 2	3 to 4	*p*
	*n* (%)	*n* (%)		*n* (%)	*n* (%)	
HBV *						
GG	16 (48.5)	2 (40.0)	0.5388 ^g^	17 (47.2)	2 (50.0)	0.8647 ^g^
GC	16 (48.5)	2 (40.0)		17 (47.2)	2 (50.0)	
CC	1 (3.0)	1 (10.0)		2 (5.6)	0 (0.0)	
G	0.73	0.60	0.0722 ^c^	0.70	0.75	0.5264 ^c^
C	0.27	0.40		0.30	0.25	
HCV **			0.1619 ^g^			0.6926 ^g^
GG	42 (79.2)	22 (61.1)		48 (72.7)	24 (70.6)	
GC	11 (21.0)	13 (36.1)		17 (25.8)	10 (29.4)	
CC	0 (0.0)	1 (2.8)		1 (1.5)	0 (0.0)	
G	0.90	0.79	0.0507 ^c^	0.74	0.85	0.0798 ^c^
C	0.10	0.21		0.26	0.15	

*n*: number of individuals; g: G test; c: chi-squared test. Patients without genotyping: * *n* = 2 and ** *n* = 1.

## Data Availability

The data analyzed in this study are included within the paper.
